# Critical Re-Examination of the Synthesis of Adamantyl Hydroperoxide

**DOI:** 10.3390/molecules31071073

**Published:** 2026-03-25

**Authors:** Ilya Nazarov, Daria Zapravdina, Anna Maximova, Ilya A. Yakushev, Victor Chapurkin, Vladimir Burmistrov

**Affiliations:** 1 Department of Organic Chemistry, Volgograd State Technical University, 400005 Volgograd, Russia; ilya.ilya.nazarov@list.ru (I.N.); zapravdinadasha94@gmail.com (D.Z.); chapurkin@vstu.ru (V.C.); 2 Laboratory of Metal Complex Catalysis, Kurnakov Institute of General and Inorganic Chemistry, Russian Academy of Sciences, 119071 Moscow, Russia; mr.albatroz@yandex.ru (A.M.); ilya.yakushev@igic.ras.ru (I.A.Y.)

**Keywords:** 1,3-dehydroadamantane, hydroperoxides, hydrogen peroxide, 1-hydroperoxyadamantane, SC XRD

## Abstract

This study investigates the synthesis of 1-hydroperoxyadamantane, addressing discrepancies in the prior literature. Our results demonstrate that previous authors were unable to obtain the claimed compound under the conditions they described. We confirmed that 1-hydroperoxyadamantane can be synthesized in good yields via the reaction of 1,3-dehydroadamantane with concentrated hydrogen peroxide in DCM. Structure of 1-hydroperoxyadamantane was confirmed by SC XRD analysis. These findings clarify the conditions necessary for successful synthesis of this compound and highlight the importance of comprehensive analytical verification. This study also provides a very detailed, repeatedly verified method for synthesizing 1,3-dehydroadamantane and a description of the necessary laboratory equipment.

## 1. Introduction

It is known that 1,3-dehydroadamantane, which belongs to the group of strained propellanes, enters into many addition reactions with various reagents [1,2,3]. Reactions of compounds containing a multiple bond with hydrogen peroxide in many cases proceed ambiguously, with the formation of a complex mixture of products that cannot always be separated [4]. The closest analogs of such a reaction include the well-studied interaction of carbonyl compounds with hydrogen peroxide, which proceeds with the formation of a complex mixture of peroxide compounds. In this reaction, hydrogen peroxide, as a nucleophilic reagent, reacts at the carbonyl group with the formation of a primary intermediate, which then interacts with the carbonyl group to form a new peroxide. Further reactions occur with the formation of a complex mixture of difficult-to-separate peroxides [5]. The interaction of unsaturated compounds with hydrogen peroxide is less studied, but in this case, a complex mixture of difficult-to-separate peroxides is also formed [6].

Isolating individual peroxides from a mixture is a virtually impossible task, as many of them are unstable compounds and decompose during isolation. New approaches to peroxide synthesis, including reactions with hydrogen peroxide, have proven to be successful. These were implemented using carbon tetrachloride or dichloroethane rather than diethyl ether, which is generally accepted for such reactions. Changing the solvent polarity previously allowed us to obtain new hydroxyhydroperoxides based on fluorinated and non-fluorinated carbonyl compounds, whether aliphatic, cyclic, or aromatic [7]. The use of diethyl ether as a solvent proved erroneous, as it facilitated the conversion of the initial hydroxyhydroperoxide reaction products into further substitution products due to the formation of hydrogen bonds between the solvent, hydrogen peroxide, and the resulting hydroxyhydroperoxide. An exception is hydroxyhydroperoxides obtained by reacting hydrogen peroxide with perfluorocyclohexanone and perfluorocyclopentanone. In this reaction, with a lack of hydrogen peroxide, 1,1-dihydroxyperoxides were formed, but they turned out to be unstable and decomposed within 24 h [8].

Thus, there are virtually no targeted attempts to synthesize 1-hydroperoxyadamantane in the literature. In a number of studies, it is cited as a minor oxidation product of adamantane derivatives, but no data on its isolation or detailed characterization are provided in these articles [9,10,11,12]. The interaction of 1,3-dehydroadamantane with hydrogen peroxide, which can lead to the formation of 1-hydroperoxyadamantane, deserves special mention. The authors of [13,14] did not pay attention to such key features of reactions involving hydrogen peroxide as the role of the solvent and the effect of temperature, which determine the possibility of peroxide formation. Widely known results of studies on the interaction of hydrogen peroxide with compounds containing multiple bonds were not analyzed, which could have made it possible to avoid a number of errors in the experimental design and to establish the absence of peroxide compounds in the reaction products. The authors of these studies reacted 1,3-dehydroadamantane with hydrogen peroxide in diethyl ether, but the latter readily forms hydrogen bonds with hydrogen peroxide and prevents the formation of 1-hydroperoxyadamantane. This reaction should have also been carried out with an excess of hydrogen peroxide, but the authors, using their own erroneous method of extracting a 30% aqueous hydrogen peroxide solution with diethyl ether, were unable to obtain highly concentrated hydrogen peroxide solutions. They also failed to perform high-quality reactions on the peroxide group in their reaction products. Therefore, the only method for synthesizing 1-hydroperoxyadamantane described in the literature requires critical rethinking.

## 2. Results and Discussion

First, we extracted an aqueous solution of hydrogen peroxide with absolute diethyl ether using the method of the authors of the patent [14] and found (by iodometric titration) that the concentration of hydrogen peroxide in the ether solution obtained in this way was 2.8 ± 0.1%. Thus, the described reaction of 1,3-dehydroadamantane with hydrogen peroxide was carried out in the absence of the latter. By repeating the described method, we indeed obtained a crude product whose heating above 200 °C was accompanied by sublimation, which the authors mistakenly took for the decomposition of 1-hydroperoxyadamantane. TLC analysis of this product for the presence of peroxide compounds (diethyl ether:cyclohexane = 1:1 system, visualization with KI in CH_3_COOH) revealed only one spot Rf = 0.36, which refers to the residual content of hydrogen peroxide. Spectral analysis confirmed that adamantane is the reaction product under the specified conditions.

**Scheme 1:**
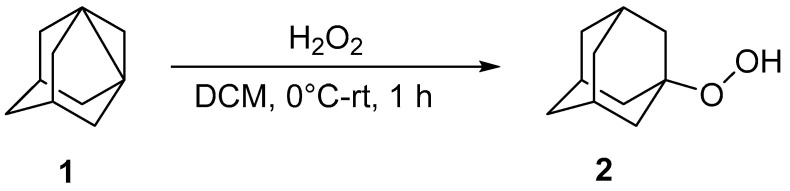
Method for the synthesis of 1-hydroperoxyadamantane (**2**).

Then we studied the reaction of 1,3-dehydroadamantane (**1**) with hydrogen peroxide using DCM as a solvent (Scheme 1). We used 90% hydrogen peroxide, to which a solution of 1,3-dehydroadamantane in DCM was added dropwise at 0–5 °C. After adding the reagents, the reaction mixture was slowly brought to room temperature and stirred for 1 h. The solvent was removed under vacuum. After recrystallization from pentane, a product with an mp of 89–91 °C was obtained in 55% yield. TLC carried out in a diethyl ether:cyclohexane = 1:1 system (visualization with KI in CH_3_COOH) showed the presence of only one peroxide with Rf = 0.7.

Under GC-MS conditions, 1-hydroperoxyadamantane (**2**) decomposes to form 1-hydroxyadamantane (**3**). Therefore, we compared the NMR spectra of the obtained product and commercially available **3**. A comparison of the chemical shifts in the ^1^H and ^13^C NMR spectra of compounds **2** and **3** is presented in Table 1. Although the differences for most protons and carbons are insignificant, a tendency for all signals to shift downfield is observed for 1-hydroperoxyadamantane (**2**). Characteristic chemical shifts are observed for protons bound to the oxygen atom (4.29 ppm for 1-hydroxyadamantane and 10.59 ppm for 1-hydroperoxyadamantane) in the NMR ^1^H spectra and carbons (66.4 ppm for 1-hydroxyadamantane and 82.8 ppm for 1-hydroperoxyadamantane) in ^13^C spectra. Such significant differences make it easy to distinguish between these two compounds. The spectrum we obtained for **3** matches the published data [15] very closely (Table 1). This allows us to conclude that the spectrum of **2**, obtained under similar conditions on the same instrument, is also reliable.

By slow evaporation of a solution of 1-hydroperoxyadamantane in a mixture of dichloromethane and ethanol (1:1) at 4 °C, crystals suitable for study by SC XRD analysis were obtained. This finally confirmed that we had obtained compound **2**.

**Figure 1:**
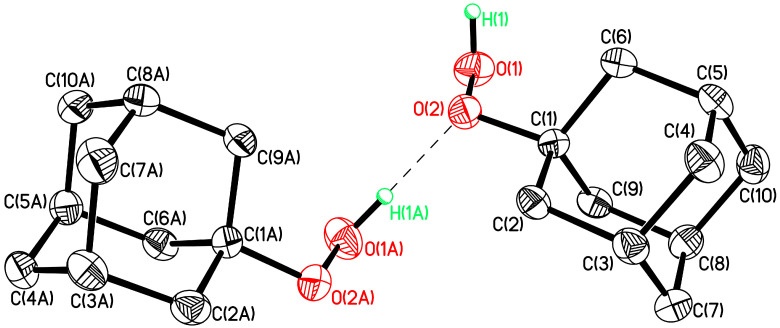
The molecular structure and atom numbering scheme of 1-hydroperoxoadamantane (**2**), with displacement ellipsoids drawn at the 50% probability level. C–H hydrogen atoms are omitted for clarity.

**Figure 2:**
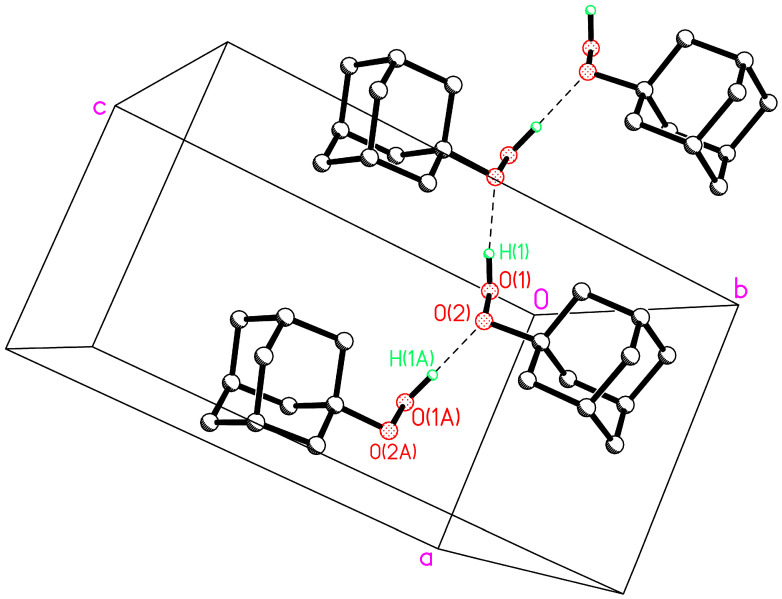
Hydrogen bonding between molecules of 1-hydroperoxoadamantane (**2**).

According to the SC XRD data, **2** crystallizes in the monoclinic *P*2_1_ space group with two crystallographically independent molecules in an asymmetric unit (Figure 1). Both molecules have similar molecular geometry with slightly different interatomic distances and angles, within the experimental error (Tables S2 and S3). At the same time, all bond lengths and angles are within normal range for the adamantane moiety, and a more noticeable feature of the molecule is its hydroperoxo group, forming intermolecular hydrogen bonds with neighboring molecules (Figure 1 and Figure 2).

The C–O (1.449(2)–1.451(2) Å) and O–O (1.469(2) Å) distances are varying within values that are close to the mean C–O and O–O bond lengths (1.43 Å and 1.46 Å, respectively) for H–O–O–C groups deposited in the Cambridge Structural Database (274 entries). It should be noted that the difference between dihedral angles C(1)-O(2)-O(1)-H(1) 101(2)° and C(1A)-O(2A)-O(1A)-H(1A) 97(3)° cannot be considered statistically significant in that case. Each molecule of **2** takes part in the formation of two intermolecular hydrogen bonds, being a donor in the case of hydrogen atoms H(1) and H(1A) and an acceptor by oxygen atoms O(2) and O(2A), and such interactions can be classified as classic-type H bonds based on geometrical parameters (Table S4); this is also confirmed and agrees with parameters of much more common hydrogen bonds in organic structures formed by hydroxyl groups with a mean distance between donor and acceptor atoms of 2.755 Å (CSD, 2930 entries for low-temperature non-disordered structures, containing only C, H, O atoms).

**Figure 3:**
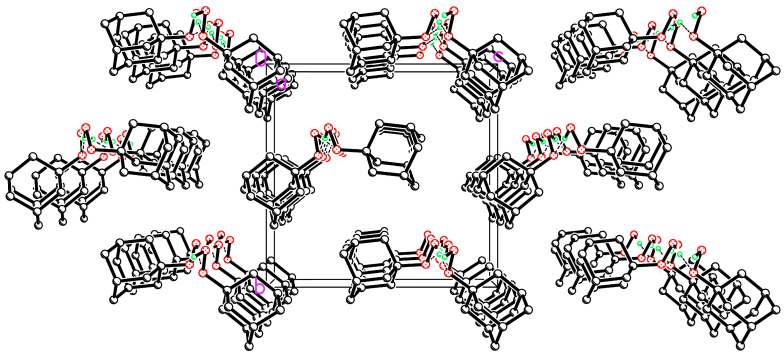
Fragment of the crystal packing of 1-hydroperoxoadamantane (**2**). Stick-and-ball model projected along the crystallographic *a*-axis. C–H hydrogen atoms are omitted for clarity.

Crystal structure packing analysis showed that molecules of **2** form infinite chains via hydrogen bonding, which in turn interact with each other through weak Van der Waals interactions (Figure 3) as expected.

When the reaction time of 1,3-dehydroadamantane with hydrogen peroxide is increased to 3 h, the yield of 1-hydroperoxyadamantane decreases to 2%. According to TLC data, the same two peroxides remain in the reaction mixture: 1-hydroperoxyadamantane (Rf = 0.7) and hydrogen peroxide (Rf = 0.36). Thus, it can be concluded that when the synthesis time is increased to 3 h, 1-hydroperoxyadamantane decomposes and is converted to adamantane.

**Scheme 2:**
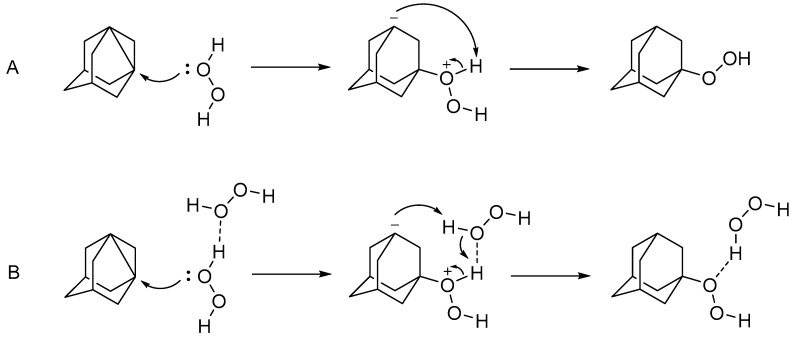
(**A**) Proposed bimolecular mechanism of interaction of **1** with hydrogen peroxide; (**B**) Alternative proposed mechanism in which H transfer is mediated by two molecules of hydrogen peroxide.

Since the reaction proceeds quite rapidly at low temperatures, we assume an ionic mechanism (Scheme 2). Option A involves an interaction between a molecule of compound **1** and one molecule of hydrogen peroxide. Although this option is possible, due to the steric rigidity of the adamantyl fragment, option B seems more likely, in which H transfer is mediated by two molecules of hydrogen peroxide.

Due to the specific shape of the propellane bond in compound **1**, two nodal carbon atoms are exposed to attack by a nucleophile. In the first step, the oxygen electron pair attacks one of the nodal atoms (either one, as they are equivalent). Both electrons from the propellane bond are then localized on the second nodal atom. In the second step, the positively charged oxygen atom takes the electrons from the O-H bond, and the corresponding hydrogen atom moves to the negatively charged nodal carbon atom, forming compound **2**.

## 3. Materials and Methods

The structure of the obtained compounds was confirmed using ^1^H and ^13^C NMR spectroscopy on a Bruker DPX 300 spectrometer (300 MHz, Bruker, Billerica, MA, USA) in DMSO-*d*_6_ solvent. NMR spectra were calibrated using residual solvent signals. Mass spectra were recorded on an Agilent MS 5977b chromatograph/mass spectrometer (Agilent Technologies, Inc., Santa Clara, CA, USA) with electron impact ionization (EI). High-resolution mass spectra were obtained on a Maxis MicroTOF II time-of-flight mass spectrometer system (Bruker Daltonics Inc., Bremen, Germany). The purity of the obtained compounds was determined on an HPLC-UV system (Jasco PU 980 pump (Tokyo, Japan), Gilson 159 detector (Middleton, WI, USA)) and Dr. Maisch Reprosil-Pur C18-AQ column (150 × 4 mm, 3 μm). Purification of the obtained compounds was performed on a Büchi Pure C-815 Flash flash chromatograph (Büchi Labortechnik AG, Flawil, Switzerland) using FlashPure cartridges. Melting points were measured on a Büchi M-565 (Büchi Labortechnik AG, Switzerland), and the average values of three independent experiments were recorded.

1,3-Dibromoadamantane was synthesized as described in reference [16]. Commercially available 30% hydrogen peroxide was concentrated to 90% in a rotary evaporator under reduced pressure and calibrated iodometrically. Other reagents were commercially available and were used without additional purification. Solvents were dried using generally known methods.

### 3.1. Synthetic Procedures

**Preparation of a catalyst for the synthesis of 1,3-dehydroadamantane.** A 50 mL flat-bottom conical flask was charged with 20 mL of anhydrous THF, 2.0 g of finely chopped lithium metal, and 150 µL of MePh_2_SiCl. The flask was purged with argon, sealed with parafilm, and stirred magnetically. The catalyst prepared in this manner can be used the next day and stored for 3 months on a shelf at room temperature without loss of activity.

**Synthesis of 1,3-dehydroadamantane (1).** First, 5.5 g of 1,3-dibromoadamantane was added to a pre-prepared catalyst. The flask was purged with argon, sealed with parafilm, and stirred on a magnetic stirrer for 3 h. After this time, the reaction mixture was decanted into a round-bottomed flask, which also contained boiling stones. The flask was placed in an oil bath, and a sublimation vessel was placed on top, connected to a vacuum. The vacuum was set to 0.1 atm and the bath temperature to 90 °C. After 15 min, boiling of the solvent ceased. The bath temperature was then raised to 130 °C, and maximum vacuum was set. Ice was then added to the sublimation vessel and maintained for 15 min. Precipitation of 1,3-dehydroadamantane was observed on the surface of the sublimation vessel. The vacuum was released, the remaining ice was removed, and the sublimation vessel was transferred to another 50 mL flask containing 20 mL of DCM. The DCM was brought to a boil and the 1,3-dehydroadamantane was washed off. Obtained 2.0 g (80% yield) as white crystalline solid. Mass spectrum, *m/z* (*I_rel_*., %): 134 (62%, [M]^+^), 119 (50%), 105 (30%), 91 (85%), 79 (100%).

Laboratory setup for the synthesis of 1,3-dehydroadamantane (**1**) is presented in the Supplementary Materials (Figure S8).

**Attention!** It is important for the reader to understand the specifics of peroxides. These compounds are chemically unstable and subject to decomposition. Any uncontrolled increase in temperature, mechanical stress, or contact with transition metals or their salts and reducing agents can lead to rapid decomposition of the substance with increased gas formation.

The following method was used to prepare 90% hydrogen peroxide: 30–50 mL of commercially available 30% hydrogen peroxide was placed in a flask and evaporated on a rotary evaporator under reduced pressure (30 mbar) and a bath temperature of 50–60 °C. Lubricating ground glass joints with vacuum grease is strictly prohibited, as hydrogen peroxide is a strong oxidizing agent. The concentration of hydrogen peroxide was monitored using the refractive index: 1.3524 for 30% and 1.3980 for 90%.

**Synthesis of 1-hydroperoxyadamantane (2).** The reactor was charged with 1.5 g (0.045 mol) of 90% hydrogen peroxide solution and cooled to 0–5 °C. Under argon atmosphere, 2.0 g (0.01 mol) of **1** dissolved in 10 mL of DCM was added dropwise with stirring. After mixing the reagents, the temperature was raised to room temperature and the mixture was stirred for 1 h. The solvent was removed under vacuum. Crude product was purified by recrystallization from pentane. Obtained 1.1 g (55% yield) as white crystalline substance. M.p. 89–91 °C. TLC in the system diethyl ether:cyclohexane = 1:1, visualization by KI in CH_3_COOH Rf = 0.7 (H_2_O_2_ in this system has Rf = 0.36). NMR ^1^H (DMSO-*d*_6_), δ, ppm: 1.54–1.61 m (6H, Ad), 1.68 d (3H, *J* 3.0 Hz, Ad), 2.10 br.s (3H, Ad), 10.60 br.s (1H, OOH). NMR ^13^C (DMSO-*d*_6_), δ, ppm: 29.7 (3C), 36.0 (3C), 39.9 (3C), 77.6 (1C, C-O). HRMS-ESI *m/z* calculated for [M + Na]^+^ C_10_H_16_NaO_2_^+^ = 191.1043, found 191.1040. Under GC-MS conditions, it decomposes to 1-hydroxyadamantane. HP-5MS quartz capillary column (30 m × 0.25 mm × 0.5 μm film thickness) in programmed temperature mode (from 80 to 280 °C), carrier gas helium, injector temperature 250 °C, quadrupole temperature 150 °C.

### 3.2. X-Ray Crystallography

The single-crystal X-ray data for **2** was collected using a Bruker D8 Venture Photon II four-circle diffractometer (Bruker, Billerica, MA, USA) in *φ*- and *ω*-scan mode (Mo *K*α radiation, λ = 0.71073 Å) at the Center for Collective Use of the Kurnakov Institute RAS (Moscow, Russia). The raw data was indexed and integrated with the *APEX*3 program suite [17]. Experimental intensities were corrected for absorption effects using *SADABS* [18]. The crystal structure was solved by direct methods [19] and refined by the full-matrix least squares on *F*^2^ [20] using the *OLEX*2 structural data visualization and analysis program suite [21]. All non-hydrogen atoms were refined with anisotropic thermal parameters. The C–H and O–H hydrogen atoms were located in difference-Fourier maps and refined isotropically. A table containing crystallographic data and structure refinement details for **2** was prepared using *pyCIFer* [22] and is given in Table S1.

Crystal data for **2**: C_10_H_16_O_2_, *M* = 168.23, *P*2_1_, *a* = 6.4481(3) Å, *b* = 11.5705(4) Å, *c* = 11.9634(4) Å, β = 90.4120(15)°, *V* = 892.54(6) Å^3^, Z = 4, *d*_calc_ = 1.252 g/cm^3^. Colorless prism single crystal with dimensions 0.19 × 0.18 × 0.14 mm was selected and intensities of 14,961 reflections were collected (*μ* = 0.085 mm^–1^, *θ*_max_ = 30.517°). After merging of equivalence reflections and absorption corrections, 5432 independent reflections (*R*_int_ = 0.0350) were used for the structure solution and refinement. Final *R* factors *R*_1_ = 0.0429 [for 4384 reflections with *F*^2^ > 2*σ*(*F*^2^)], *wR*_2_ = 0.0993 (for all reflections), *S* = 1.024, and largest diff. peak and hole are 0.209 and −0.181 e/Å^3^, respectively.

## 4. Conclusions

In the present work, we demonstrated that 1-hydroperoxyadamantane can be obtained in good yields via the reaction of 1,3-dehydroadamantane with concentrated hydrogen peroxide in dichloromethane. Thus, we assume that the authors of previous studies were unable to obtain either 1-hydroperoxyadamantane or diadamantyl peroxide under the conditions they described. The ^1^H NMR signal reported by them as a broad singlet at 7.5 ppm likely corresponds to residual hydrogen peroxide. In contrast, the spectra of 1-hydroperoxyadamantane crystals, obtained by us, display a broad singlet for the hydroperoxide group proton with a chemical shift of 10.60 ppm. Moreover, the method of synthesizing 1,3-dehydroadamantane that we have described in detail allows anyone to reproduce it to obtain hard-to-obtain adamantane derivatives.

## Figures and Tables

**Table 1:** Comparison of chemical shifts in NMR spectra for 1-hydroperoxyadamantane (**2**) and 1-hydroxyadamantane (**3**).

	^1^H				^13^C			
	4, 8, 10 CH_2_	2, 6, 9 CH_2_	3, 5, 7 CH	OH	4, 8, 10 CH_2_	2, 6, 9 CH_2_	3, 5, 7 CH	1 C-O
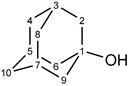	1.51–1.56 m (1.54 br) [15]	1.58 d 2.9 Hz (1.58 d 2.5 Hz) [15]	2.03 s (2.04 s) [15]	4.29 s (4.28 s) [15]	30.5	36.4	45.7	66.4
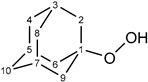	1.54–1.61 m	1.68 d 3.0 Hz	2.10 br.s	10.59 br.s	29.7	36.0	39.9	77.6

## Data Availability

The data underlying this study are available in the published article and its Supplementary Materials.

## Supplementary Materials

The following supporting information can be downloaded at https://www.mdpi.com/article/10.3390/molecules31071073/s1. Figure S1. Chromatogram of the reaction mass containing 1,3-dehydroadamantane (95%, retention time 6.086 min) with 4% of adamantane impurity (retention time 6.607 min). Figure S2. Mass spectra of 1,3-dehydroadamantane (**1**). Figure S3. NMR ^1^H of 1-hydroperoxyadamantane (**2**). Figure S4. NMR ^13^C of 1-hydroperoxyadamantane (**2**). Figure S5. HRMS (ESI) of 1-hydroperoxyadamantane (**2**). Figure S6. NMR ^1^H of adamantan-1-ol (AdOH). Figure S7. NMR ^13^C of adamantan-1-ol (AdOH). Figure S8. Laboratory setup for the synthesis of 1,3-dehydroadamantane. Table S1. Crystallographic data and structure refinement parameters for **2**. Table S2. Selected bond lengths for **2**. Table S3. Selected angles for **2**. Table S4. Hydrogen bonds for **2** [Å and angles]. Accession Codes: Deposition Number 2487732 contains the supplementary crystallographic data for this paper. These data are provided free of charge by The Cambridge Crystallographic Data Centre: ccdc.cam.ac.uk/structures. The corresponding CIF files are also available as Supplementary Materials.
