# Supplementary material for: Critical Re-Examination of the Synthesis of Adamantyl Hydroperoxide
Source: Molecules. 2026 Mar 25;31(7):1073. doi: 10.3390/molecules31071073 (PMC13075001; doi:10.3390/molecules31071073)
Supplement: Supplementary file 1 [file molecules-31-01073-s001.zip › molecules-4193458-supplementary.pdf]

Critical Re-Examination of the Synthesis of Adamantyl Hydroperoxide

Ilya Nazarov [1], Daria Zapravdina [1], Anna Maksimova [2], Ilya Yakushev [2], Victor Chapurkin [1], and Vladimir Burmistrov [1]

[1] Department of Organic Chemistry, Volgograd State Technical University, Volgograd, 400005, Russia; vburmistrov@vstu.ru (V.B.)
[2] Laboratory of metal complex catalysis, Kurnakov Institute of General and Inorganic Chemistry, Russian Academy of Sciences, Moscow, 119071, Russia; ilya.yakushev@igic.ras.ru (I.Y.)

Supplementary materials

Spectral and SC XRD data

Figure S1. Chromatogram of the reaction mass containing 1,3-dehydroadamantane (95%, retention time 6.086 min) with 4% of adamantane impurity (retention time 6.607 min)

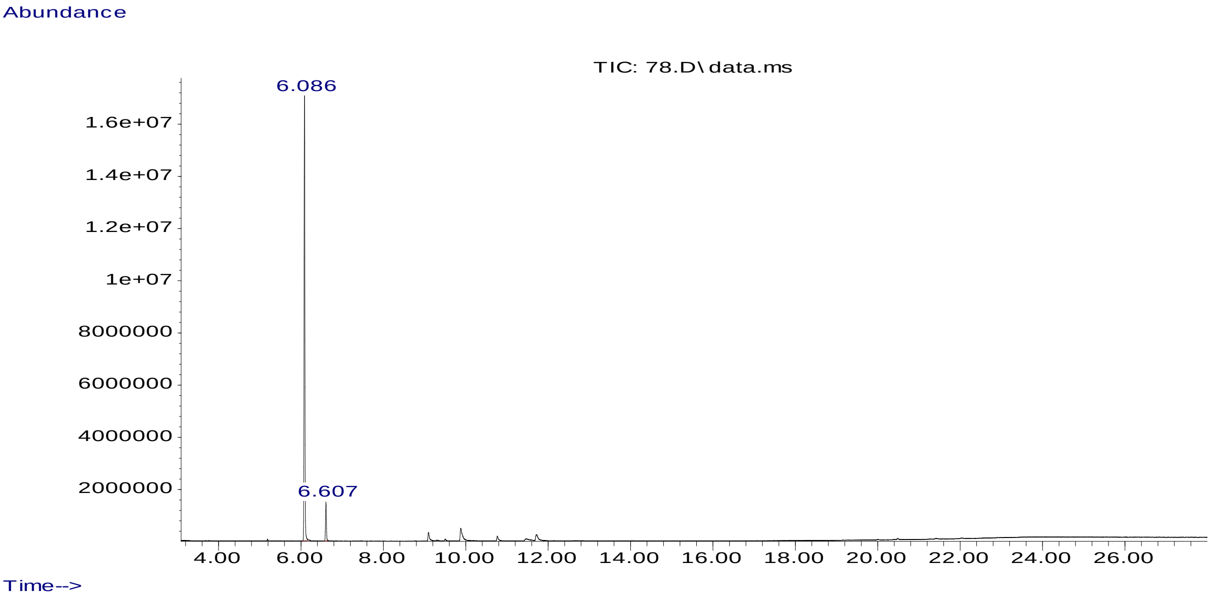

Figure S2. Mass-spectra of 1,3-dehydroadamantane (**1**)

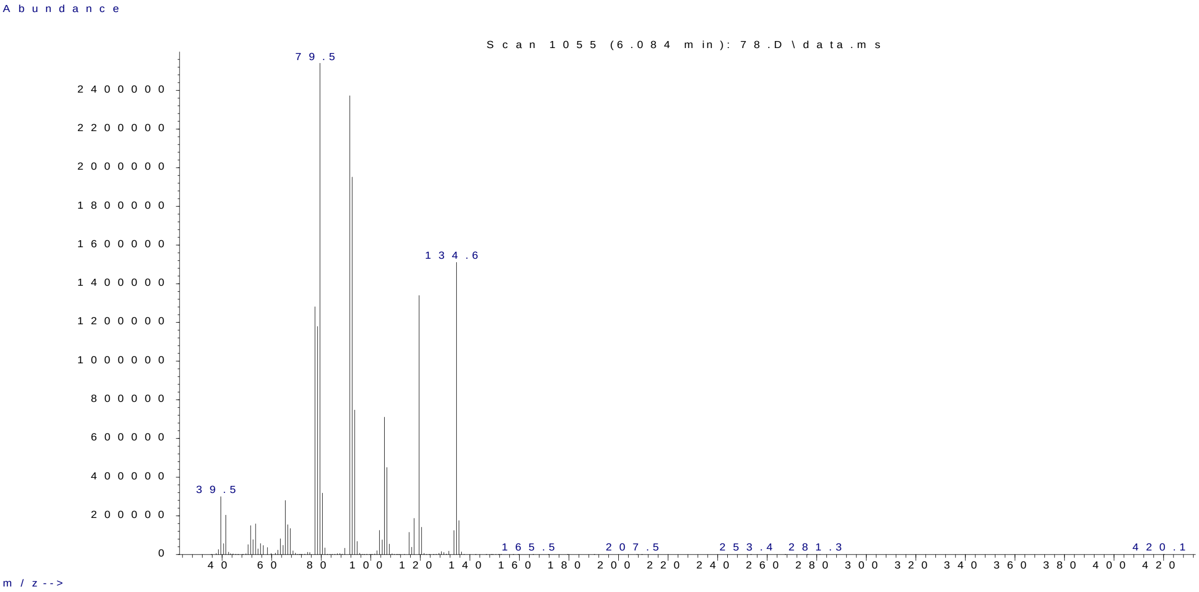

Figure S3. NMR ^{1}H of 1-hydroperoxyadamantane (**2**)

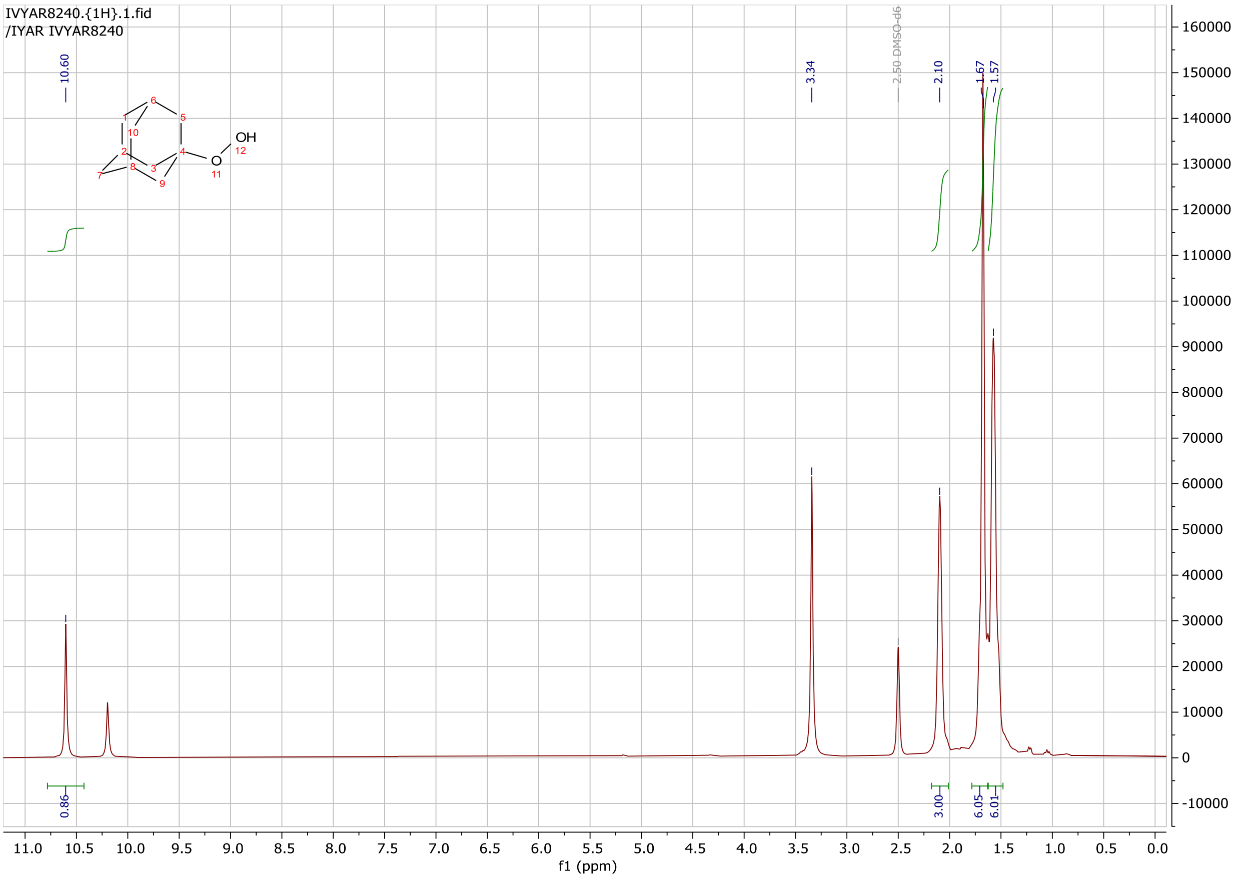

Figure S4. NMR ^{13}C of 1-hydroperoxyadamantane (**2**)

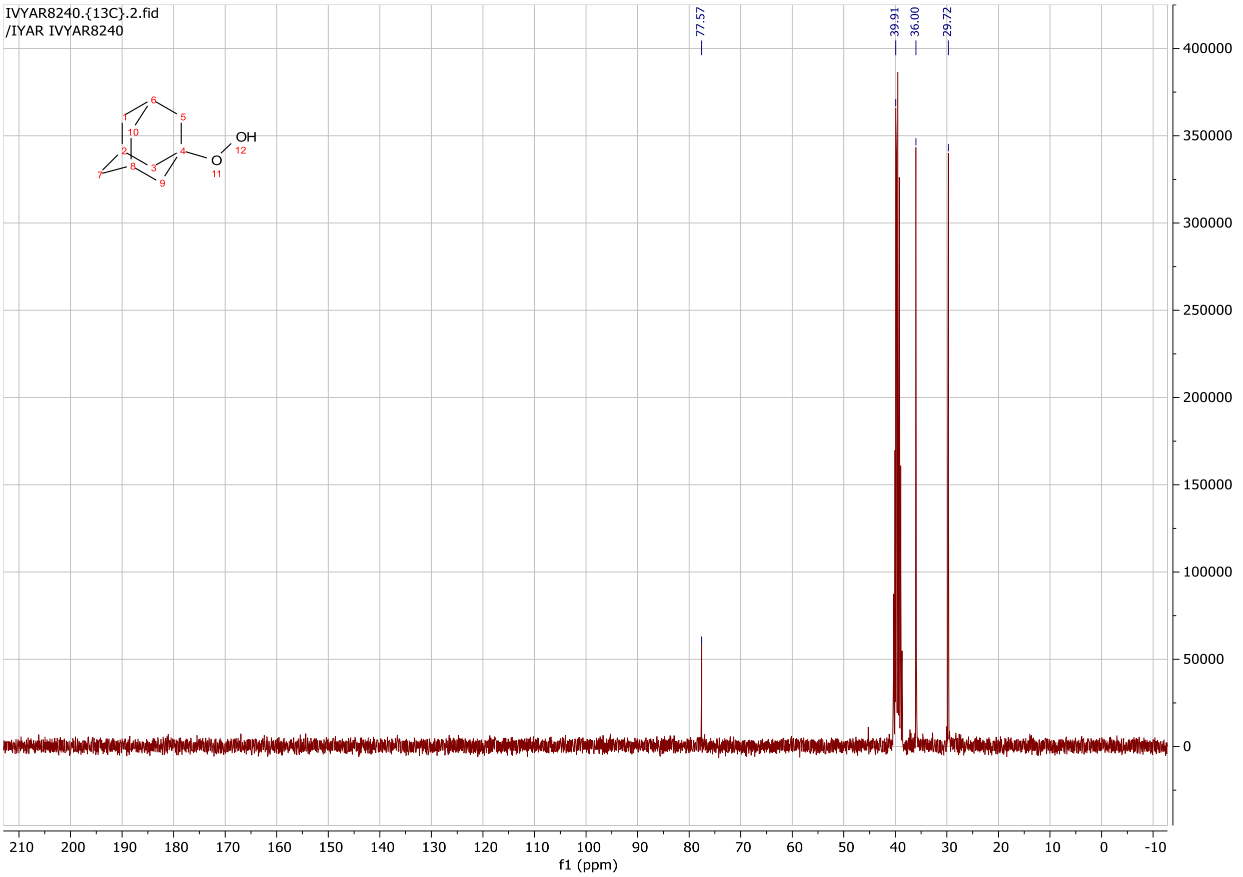

Figure S5. HRMS (ESI) of 1-hydroperoxyadamantane (**2**)

Display Report

Analysis Info

Analysis Name	D:\Data\Kolotyrkina\2025\Dmitrieva\1008029.d	Acquisition Date	08.10.2025 16:10:33
Method	tune_100-1200.m	Operator	BDAL@DE
Sample Name	/IYAR AdOOHs	Instrument / Ser#	maXis 43
Comment	C10H16O2 clb added CH3OH		

Acquisition Parameter

Source Type	ESI	Ion Polarity	Positive	Set Nebulizer	1.0 Bar
Focus	Active			Set Dry Heater	200 °C
Scan Begin	50 m/z	Set Capillary	4500 V	Set Dry Gas	4.0 l/min
Scan End	1800 m/z	Set End Plate Offset	-500 V	Set Divert Valve	Waste

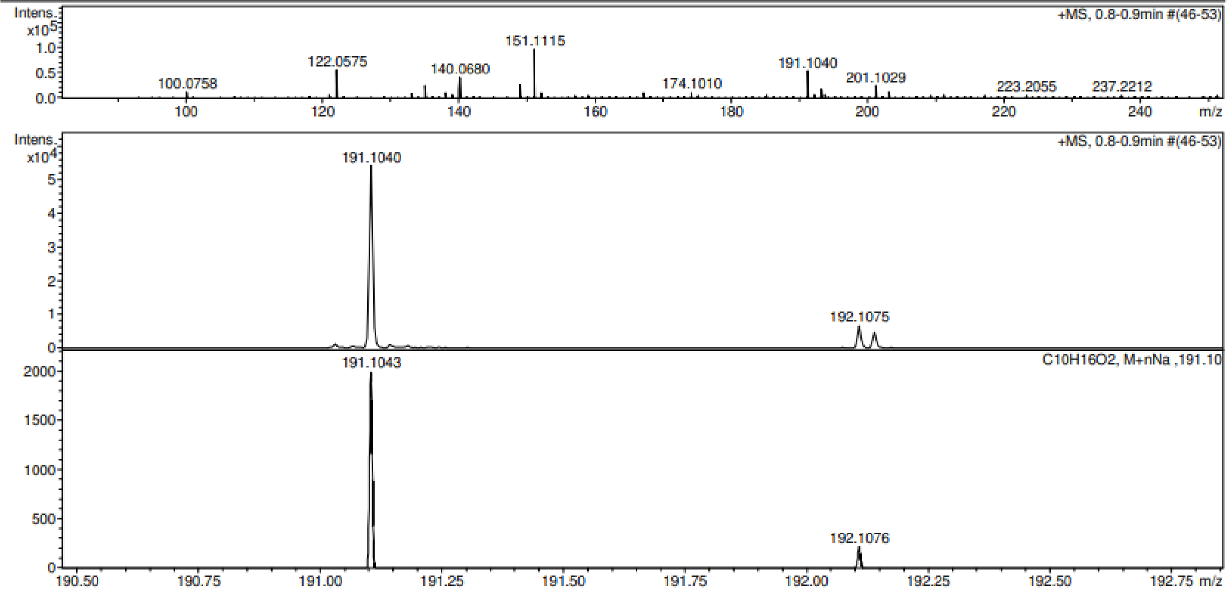

Figure S6. NMR ^{1}H of adamantan-1-ol (AdOH)

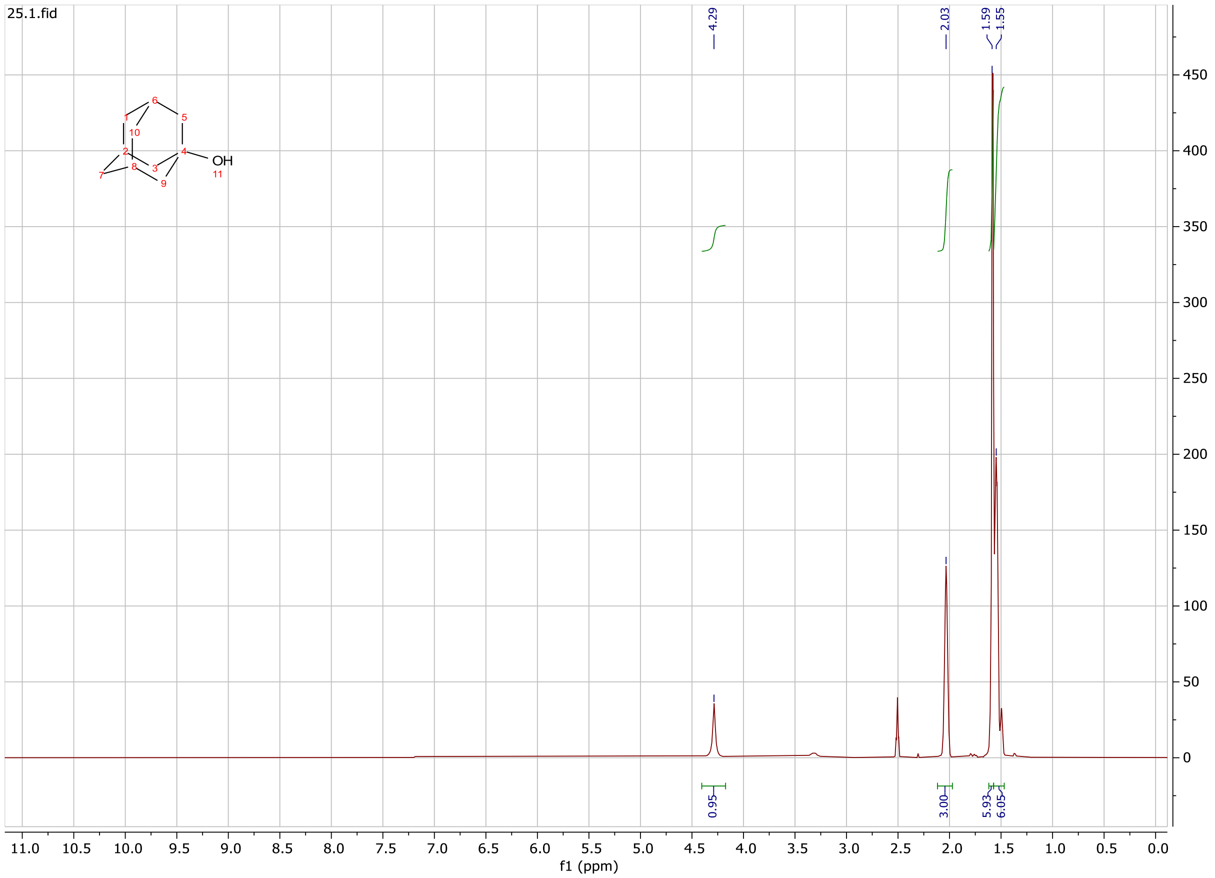

Figure S7. NMR ^{13}C of adamantan-1-ol (AdOH)

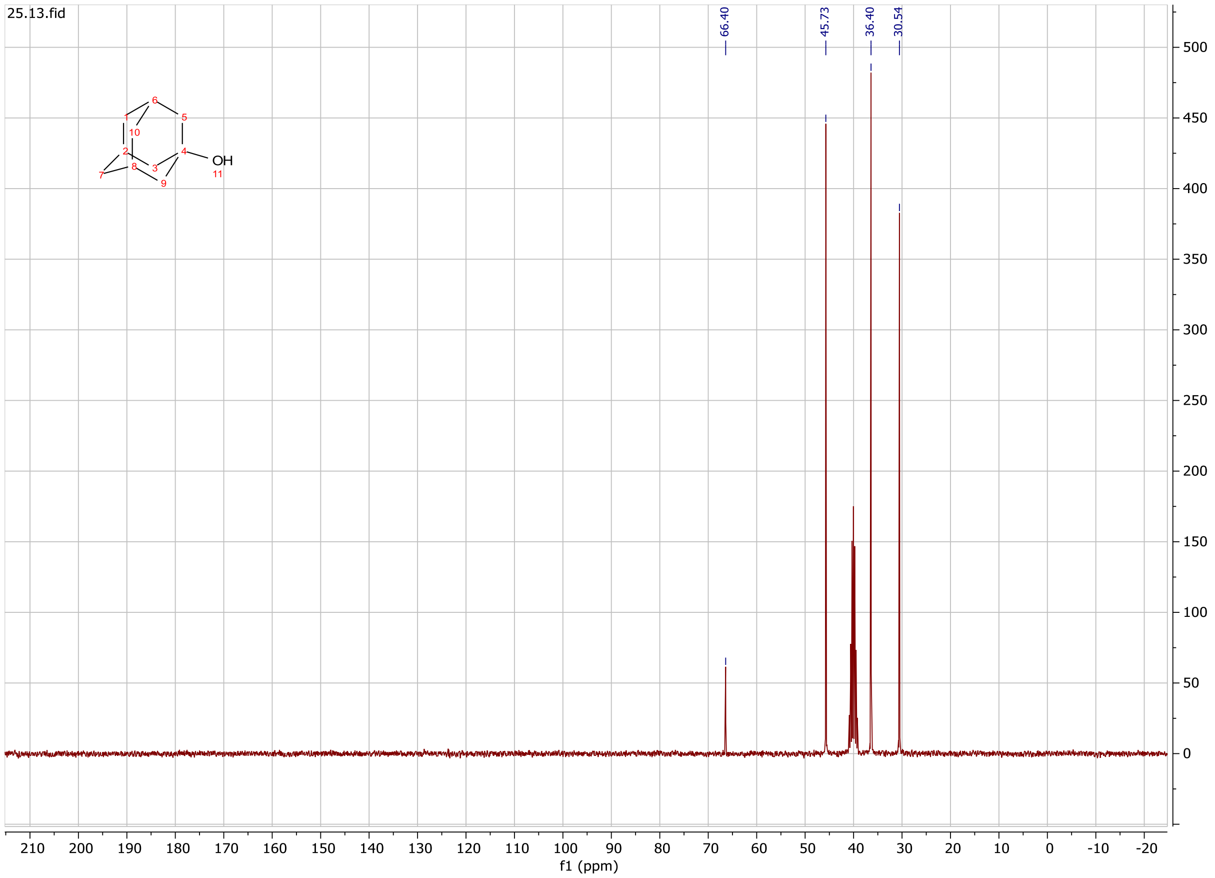

Figure S8. Laboratory setup for the synthesis of 1,3-dehydroadamantane

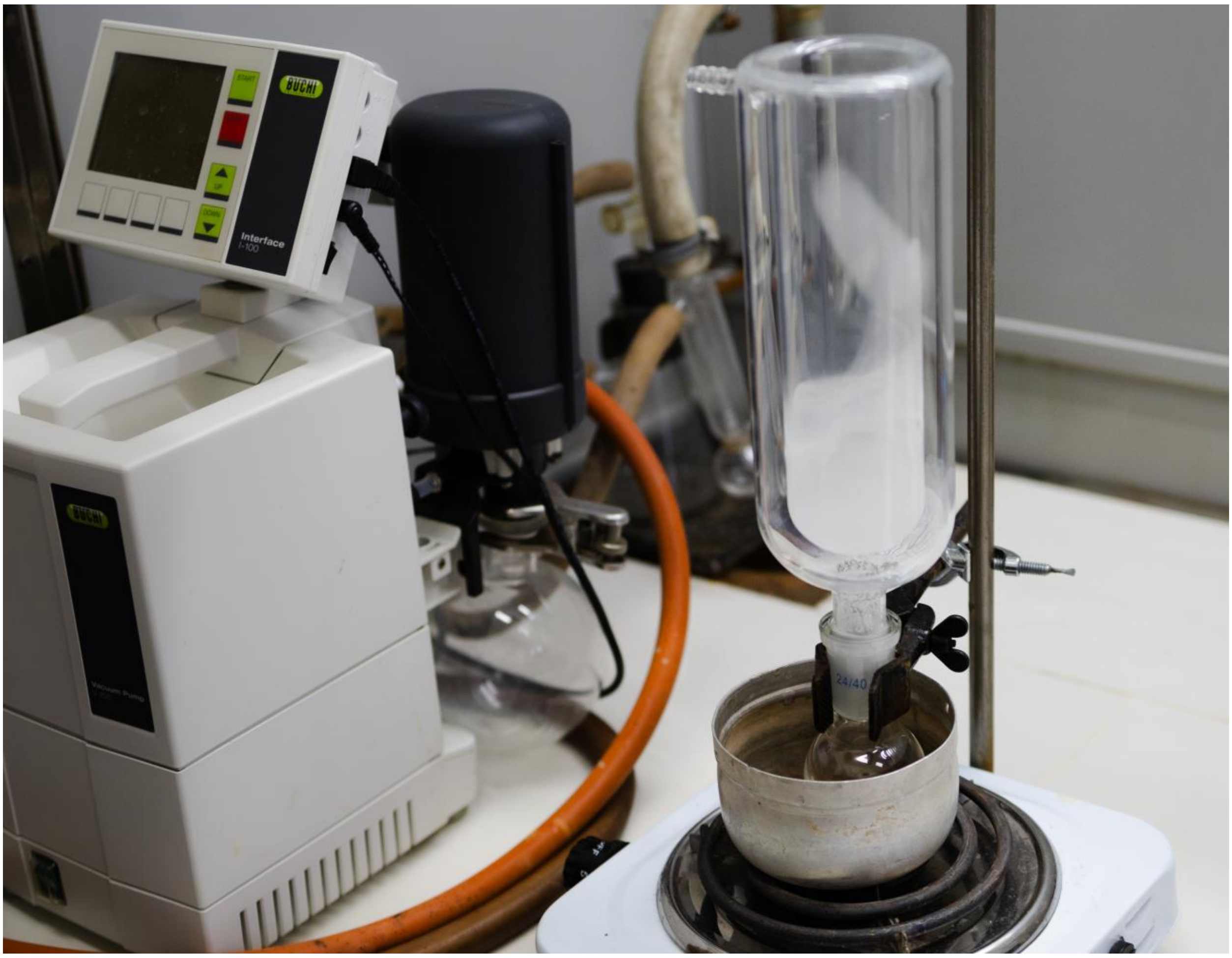

Table S1. Crystallographic data and structure refinement parameters for **2**

CCDC No.	CCDC 2487732
Empirical formula	$C_{10}H_{16}O_2$
F.W.	168.23
Colour, habit	colourless prism
Crystal size, mm	0.190 × 0.180 × 0.140
Temperature, K	150(2)
Wavelength, Å	0.71073
Crystal system	monoclinic
Space group	$P2_1$
a, Å	6.4481(3)
b, Å	11.5705(4)
c, Å	11.9634(4)
α, deg.	90
β, deg.	90.4120(15)
γ, deg.	90
V, Å^3	892.54(6)
Z	4
Density (calc.), g/cm^3	1.252
μ, mm^{-1}	0.085
F(000)	368
Theta range, deg.	1.702 – 30.517
Index ranges	$-9 \leq h \leq 9$<br>$-16 \leq k \leq 16$<br>$-17 \leq l \leq 17$
Reflections collected	14961
Independent reflections	5432 (R_{int} = 0.0350)
T_{min} / T_{max}	0.6719 / 0.7461
Data / restraints / parameters	5432 / 1 / 345
Goodness-of-fit	1.024
R_1 / wR_2 ($I > 2\sigma(I)$)	R_1 = 0.0429<br>wR_2 = 0.0922
R_1 / wR_2 (all data)	R_1 = 0.0559<br>wR_2 = 0.0993
$\Delta\rho_{max}$ / $\Delta\rho_{min}$, e·Å^{-3}	0.209 / -0.181

Table S2. Selected bond lengths for **2**

Bond	Length (Å)	Bond	Length (Å)
O(1)-O(2)	1.469(2)	O(1A)-O(2A)	1.469(2)
O(2)-C(1)	1.451(2)	O(2A)-C(1A)	1.449(2)
C(1)-C(9)	1.518(3)	C(1A)-C(6A)	1.525(3)
C(1)-C(6)	1.527(3)	C(1A)-C(9A)	1.529(3)
C(1)-C(2)	1.527(3)	C(1A)-C(2A)	1.529(3)
C(2)-C(3)	1.531(3)	C(2A)-C(3A)	1.534(3)
C(3)-C(7)	1.533(3)	C(3A)-C(4A)	1.528(3)
C(3)-C(4)	1.542(3)	C(3A)-C(7A)	1.535(3)
C(4)-C(5)	1.531(3)	C(4A)-C(5A)	1.527(3)
C(5)-C(10)	1.527(3)	C(5A)-C(10A)	1.530(3)
C(5)-C(6)	1.532(3)	C(5A)-C(6A)	1.530(3)
C(7)-C(8)	1.531(3)	C(7A)-C(8A)	1.530(3)
C(8)-C(10)	1.532(3)	C(8A)-C(10A)	1.532(3)
C(8)-C(9)	1.533(3)	C(8A)-C(9A)	1.533(3)

Table S3. Selected angles for **2**

Bond	Angle	Bond	Angle
C(1)-O(2)-O(1)	109.53(13)	C(1A)-O(2A)-O(1A)	109.46(15)
O(2)-C(1)-C(9)	111.83(16)	O(2A)-C(1A)-C(6A)	111.43(17)
O(2)-C(1)-C(6)	111.56(15)	O(2A)-C(1A)-C(9A)	111.29(16)
C(9)-C(1)-C(6)	110.52(16)	C(6A)-C(1A)-C(9A)	110.60(18)
O(2)-C(1)-C(2)	103.09(14)	O(2A)-C(1A)-C(2A)	103.70(16)
C(9)-C(1)-C(2)	109.72(16)	C(6A)-C(1A)-C(2A)	109.97(17)
C(6)-C(1)-C(2)	109.87(16)	C(9A)-C(1A)-C(2A)	109.64(18)
C(1)-C(2)-C(3)	108.89(16)	C(1A)-C(2A)-C(3A)	108.85(17)
C(2)-C(3)-C(7)	109.56(17)	C(4A)-C(3A)-C(2A)	109.82(19)
C(2)-C(3)-C(4)	109.54(18)	C(4A)-C(3A)-C(7A)	109.4(2)
C(7)-C(3)-C(4)	109.23(17)	C(2A)-C(3A)-C(7A)	108.99(19)
C(5)-C(4)-C(3)	109.08(17)	C(5A)-C(4A)-C(3A)	109.75(18)
C(10)-C(5)-C(4)	109.74(19)	C(4A)-C(5A)-C(10A)	109.78(19)
C(10)-C(5)-C(6)	109.61(18)	C(4A)-C(5A)-C(6A)	108.95(18)
C(4)-C(5)-C(6)	109.59(17)	C(10A)-C(5A)-C(6A)	109.41(17)
C(1)-C(6)-C(5)	108.72(16)	C(1A)-C(6A)-C(5A)	109.03(18)
C(8)-C(7)-C(3)	109.57(18)	C(8A)-C(7A)-C(3A)	109.44(19)
C(7)-C(8)-C(10)	109.67(18)	C(7A)-C(8A)-C(10A)	109.43(18)
C(7)-C(8)-C(9)	108.91(18)	C(7A)-C(8A)-C(9A)	109.98(19)
C(10)-C(8)-C(9)	109.27(18)	C(10A)-C(8A)-C(9A)	109.09(19)
C(1)-C(9)-C(8)	109.39(16)	C(1A)-C(9A)-C(8A)	108.57(17)
C(5)-C(10)-C(8)	109.59(17)	C(5A)-C(10A)-C(8A)	109.72(19)

Table S4. Hydrogen bonds for **2** [Å and angles]

D–H...A	*d*(D–H)	*d*(H...A)	*d*(D...A)	<(DHA)
O(1A)-H(1A)...O(2)	0.96(5)	1.80(5)	2.765(2)	175(4)
O(1)-H(1)...O(2A)[#1]	0.92(4)	1.87(4)	2.787(2)	174(3)

Symmetry transformations used to generate equivalent atoms: [#1] 1 x-1,y,z
